# Treatment strategies to prevent or mitigate the outcome of postpancreatectomy hemorrhage: a review of randomized trials

**DOI:** 10.1097/JS9.0000000000000876

**Published:** 2023-11-16

**Authors:** Roberto M. Montorsi, Babs M. Zonderhuis, Freek Daams, Olivier R. Busch, Geert Kazemier, Giovanni Marchegiani, Giuseppe Malleo, Roberto Salvia, Marc G. Besselink

**Affiliations:** a Department of Surgery, Amsterdam UMC, location University of Amsterdam, Amsterdam, the Netherlands; b Department of Surgery, Amsterdam UMC, Vrije Universiteit, Amsterdam, the Netherlands; c Cancer Center Amsterdam, De Boelelaan, Amsterdam, the Netherlands; d Department of General and Pancreatic Surgery, The Pancreas Institute, University of Verona Hospital Trust, Verona, Italy; e Department of Surgical, Hepato-Pancreato-Biliary Surgery and Liver Transplantation Unit, Oncological and Gastroenterological Sciences (DISCOG), University of Padua, Padua, Italy

**Keywords:** pancreatic surgery, postpancreatectomy hemorrhage, systematic review

## Abstract

**Background:**

Postpancreatectomy hemorrhage (PPH) is a leading cause for surgical mortality after pancreatic surgery. Several strategies for the prevention and management of PPH have been studied in randomized controlled trials (RCTs) but a systematic review is lacking. The authors systematically reviewed RCTs regarding the impact of treatment strategies on the incidence and outcome of PPH.

**Material and methods:**

Eligible RCTs reporting on impact of treatment on the rate of PPH were identified through a systematic literature search using the Evidence Map of Pancreatic Surgery (2012–2022). Methodological quality was assessed using the Cochrane Risk of Bias 2 (RoB-2) tool for RCTs. Various definitions of PPH were accepted and outcome reported separately for the International Study Group for Pancreatic Surgery (ISGPS) definition.

**Results:**

Overall, 99 RCTs fulfilled the eligibility criteria with a pooled 6.1% rate of PPH (range 1–32%). The pooled rate of PPH defined as ISGPS grade B/C was 8.1% (range 0–24.9%). Five RCTs reported five strategies that significantly reduced the rate of PPH. Three concerned surgical technique: pancreatic anastomosis with small jejunal incision, falciform ligament wrap around the gastroduodenal artery stump, and pancreaticojejunostomy (vs pancreaticogastrostomy). Two concerned perioperative management: perioperative pasireotide administration, and algorithm-based postoperative patient management. No single RCT specifically focused on the treatment of patients with PPH.

**Conclusion:**

This systematic review of RCTs identified five strategies which reduce the rate of PPH; three concerning intraoperative surgical technique and two concerning perioperative patient management. Future studies should focus on the treatment of patients with PPH as RCTs are currently lacking.

## Introduction

Highlights

- Postpancreatectomy hemorrhage (PPH) is a leading cause of surgical mortality after pancreatic surgery.
- Systematic review of randomized controlled trials regarding treatment strategies to reduce the incidence and improve outcome of PPH.
- In 43 randomized controlled trials, the pooled rate of clinically-relevant (ISGPS B/C) PPH was 8.1%.
- Five strategies reduced the rate of PPH: small jejunal incision when performing an end-to-side duct-to-mucosa pancreaticojejunostomy, a falciform ligament patch around hepatic artery and stump of gastroduodenal artery during pancreatoduodenectomy, performing pancreaticojejunostomy instead of pancreaticogastrostomy, administration of perioperative pasireotide instead of hydrocortisone and a multimodal algorithm for the postoperative management of pancreatic patients.

Pancreatic surgery remains characterized by high rates of morbidity and mortality due to its surgical complexity and high rate of postoperative pancreatic fistula (POPF). Unlike postoperative mortality, which has now decreased to less than 5% in high-volume centers, the incidence of postoperative morbidity remains in excess of 50%^1^.

Postpancreatectomy hemorrhage^1^ (PPH), POPF^2^, delayed gastric emptying^3^, chyle leak^4^, bile leak^5^ and postpancreatectomy acute pancreatitis^6^ are the most frequent and relevant complications. Among these PPH is associated with the highest mortality rate, of up to 40% in clinically severe scenarios^7^.

In 2007, the International Study Group for Pancreatic Surgery (ISGPS) published the definition of PPH in order to standardize and improve research regarding this topic. ISGPS grades PPH based on timing, location, and severity (Grades A–C). Prevention and management of PPH are a topic of considerable concern. Randomized controlled trials (RCTs) represent the best tool for obtaining strong data with the lowest risk of bias. However, a systematic review of RCTs focusing on the impact of surgical and perioperative treatment on PPH is currently lacking.

This systematic review aims to include all RCTs published in the last decade in the field of pancreatic surgery, which reported a positive or negative impact on the rate and outcome of PPH.

## Material and methods

The systematic review protocol was registered with the International Prospective Register of Systematic Reviews^8^ (PROSPERO) on 23 March 2023 and was last updated on 31 March 2023. The protocol of this systematic review was developed according to the PRISMA-P guidelines^9^. The present systematic review was structured in accordance with the Preferred Reporting Items for Systematic Reviews and Meta-Analyses (PRISMA) (Supplemental Digital Content 1, http://links.lww.com/JS9/B316) and Assessing the methodological quality of systematic reviews (AMSTAR) (Supplemental Digital Content 2, http://links.lww.com/JS9/B317) guidelines^10,11^.

### Eligibility criteria

This systematic review is based on the Evidence Map of Pancreatic Surgery, which includes all RCTs related to pancreatic surgery published between 1 January 2012 and 31 December 2022 (11 years period).^12^ All RCTs reporting on PPH (any definition) were screened and included in the present systematic review, prospective and retrospective studies, trial protocols, systematic reviews, meta-analyses, conference abstracts, secondary publications of previously published studies, letters, and commentaries were excluded. Publications without an available full-text or in languages other than English were excluded as well.

### Information sources and search strategy

A systematic literature search was performed in Evidence Map of Pancreatic Surgery^12^. The search included all RCTs related to pancreatic surgery.

### Data collections process

Two reviewers (R.M.M. and B.M.Z.) independently screened the full-text literature using the above-mentioned eligibility criteria. Disagreements were resolved by consensus and, if necessary, by the opinion of a third reviewer (M.G.B.).

### Data items

Data extraction included: publication details (e.g. study title, publication date, authors, and study design), baseline characteristics (e.g. number of patients, sex, age, and diagnosis), clinical characteristic and intervention characteristics (e.g. noninvasive treatment, endoscopic treatment, angiographic treatment, and surgical treatment) and postoperative characteristics (e.g. pancreas-specific postoperative complications, general postoperative complications, and Clavien–Dindo classification^13^). Primary and secondary endpoints were extracted additionally.

### Outcomes

Primary outcome was the rate and outcome of PPH (any definition). Secondary outcomes included various strategies of prevention and management of PPH and the rate and outcomes for the different grades of PPH (ISGPS type A, B, and C)^1^. An effective strategy for prevention of PPH was defined by a significant reduction in either all grades PPH or only grade B/C PPH.

### Study risk of bias assessment

The quality of the included studies was assessed by two independent reviewers (R.M.M. and B.M.Z.) using the Cochrane Risk of Bias 2 (RoB-2)^14^ tool for RCTs.

### Statistical analysis

Descriptive statistics were used to summarize the extracted data. Continuous data were presented as mean with SD or as median with interquartile range accordingly. Binary or categorical data were presented as frequencies (%).

### Theory

Before the publication of the PPH definition by the ISGPS in 2007, incidence and severity of PPH reported in literature varied considerably, making any comparison unreliable.

PPH is classified as early (<24 h) or late (>24 h), intraluminal or extra-luminal and mild (small volume blood loss/mild clinical impairment/no need of invasive treatment) or severe (large volume blood loss/clinically severe impairment/need on invasive treatment).

Due to its correlation with adverse postoperative course, prevention and management of PPH are continuously investigated. However, prevention and management of this life-threatening complication are still complex and much bigger efforts are needed in order to mitigate it.

## Results

### Study selection and publication details

**Figure 1:**
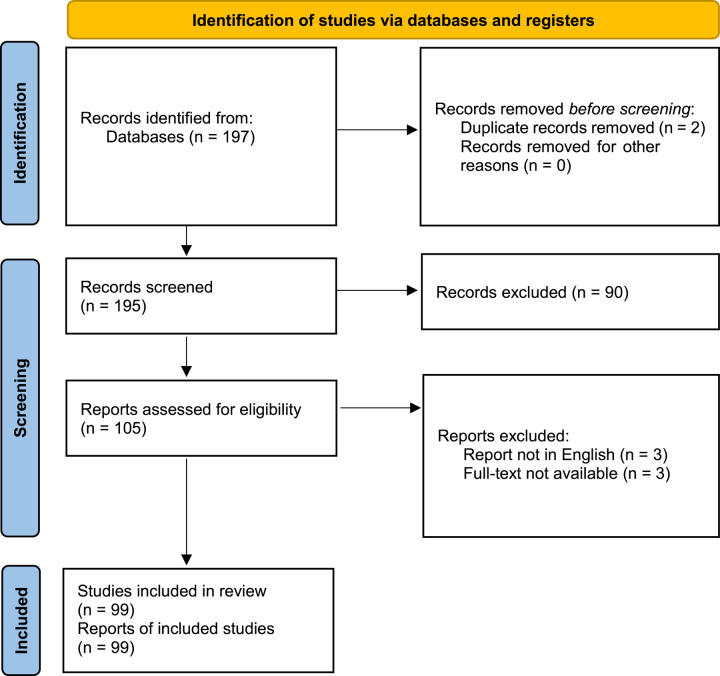
PRISMA Flow diagram.

The literature search on Evidence Map of Pancreatic Surgery identified 197 RCTs. (Fig. 1).

The first screening was based on the time of publication. Two duplicate studies^15,16^ were excluded. Hereafter, 90 RCTs were excluded because no data on PPH was reported. Three RCTs were excluded because written in a language other than English. Three RCTs were excluded because full-text was not available. Finally, 99 RCTs^15–113^ were included in the present systematic review (Tables 1, 2, 3, 4 and Supplementary Table 1, Supplemental Digital Content 3, http://links.lww.com/JS9/B318).

**Table 1:** Included randomized controlled trials on pancreatoduodenectomy.

Evidence map section	Evidence map subsection	Evidence map sub-subsection	Number of RCTs
Partial pancreatoduodenectomy	Pancreatic anastomosis	Stenting	5
		Techniques	8
		Additional interventions	2
		Pancreaticogastrostomy vs pancreaticojejunostomy	6
	Entero-enteric anastomosis	Pylorus-preserving versus pylorus-resecting	2
		Pylorus-preserving versus classical Whipple procedure	1
		Antecolic versus retrocolic gastroenteric anastomosis	4
		Billroth II vs Roux-En-Y	1
		Braun entero-enterostomy	3
		Other anastomotic techniques	1
	Drainage	Intra-abdominal drainage versus no drainage	2
		Early versus late removal of intra-abdominal drainage	3
		Type of drainage	1
	Surgical aspects	Extended versus standard resection	4
		Surgical approach	1
		Isolated Roux-En-Y pancreatojejunostomy	2
		Energy device in dissection	3
	Minimally-invasive surgery	Minimally-invasive versus open pancreatoduodenectomy	5

RCTs, randomized controlled trials.

**Table 2:** Included randomized controlled trials on distal pancreatectomy.

Evidence map section	Evidence map subsection	Evidence map sub-subsection	Number of RCTs
Distal pancreatectomy	Pancreatic remnant	Reinforced staplers	3
		Anastomosis	3
		Autologous coverage	1
		Sealants	4
	Drainage	Intra-abdominal drainage versus no drainage	1
		Early versus late removal of intra-abdominal drainage	1
		Type of drainage	1
	Surgical aspects	Spleen management	1
		Energy device dissection	1
	Minimally-invasive surgery	Minimally-invasive versus distal pancreatectomy	2

RCTs, randomized controlled trials.

**Table 3:** Included randomized controlled trials on various perioperative interventions.

Evidence map section	Evidence map subsection	Evidence map sub-subsection	Number of RCTs
Various perioperative interventions	Prevention of surgical site infection	—	4
	Intervention to improve recovery	—	4
	Perfusion management	—	2
	Other intervention to improve outcomes	—	5
	Pharmaceutical co-treatment:	Somatostatin analogs	3
		Corticosteroids	2
	Nutrition	Enriched versus standard diet	2
		Route of nutrition	3
		Time point and duration of nutritional support	1

RCTs, randomized controlled trials.

**Table 4:** Included randomized controlled trials on other surgical aspects.

Evidence map section	Evidence map subsection	Evidence map sub-subsection	Number of RCTs
Other surgical aspects	Treatment of complications	—	2
	Parenchyma-sparing interventions	—	1
	Total versus pancreatoduodenectomy	—	1

RCTs, randomized controlled trials.

The 99 RCTs were published from 24 countries and 44 were multicenter trials. Among them, 42 were nationwide multicenter RCTs and 2 were international multicenter RCTs. Samples sizes varied from 25^99^ to 1748^98^ patients. Overall, 40 RCTs referred to PPH with different definitions (e.g. bleeding, gastrointestinal hemorrhage, gastrointestinal bleeding, etc.) while 67 referred to any event of bleeding as PPH. The ISGPS definition of PPH^1^ was cited by 43 RCTs.

Overall, 53 RCTs focused on pancreatoduodenectomy (PD) (Supplementary Table 2, Supplemental Digital Content 4, http://links.lww.com/JS9/B319). Among them, 20 RCTs focused on the pancreatic anastomosis, 12 on entero-enteric anastomosis, 6 on drainage, 10 on surgical aspects, and 5 on minimally-invasive pancreatoduodenectomy.

Overall, 18 RCTs focused on distal pancreatectomy (DP) (Supplementary Table 3, Supplemental Digital Content 5, http://links.lww.com/JS9/B320). Among them, 11 RCTs focused on pancreatic remnant management, 3 on drain management, 2 on surgical aspects, and 2 on minimally-invasive distal pancreatectomy.

Overall, 26 RCTs focused on various perioperative interventions (Supplementary Table 4, Supplemental Digital Content 6, http://links.lww.com/JS9/B321). Among them, four RCTs focused on surgical site infections, four on interventions to improve recovery, two on perfusion management, five on other interventions to improve outcomes, five on pharmaceutical co-treatments, and six on nutrition.

Overall, four RCTs focused on other surgical aspects (Supplementary Table 5, Supplemental Digital Content 7, http://links.lww.com/JS9/B321). Among them, two focused on treatment of complications, one on parenchyma-sparing interventions and one on total pancreatectomy versus PD.

Among the 9986 patients treated in the included RCTs which reported the ISGPS classification, the rate of PPH varied from less than 1–32% with a mean of 6.1%. Further analysis showed that the rate of PPH ISGPS grade A varied from 0 to 10.5% and a mean of 1.7% while the rate of ISGPS PPH B/C varied from 0 to 24.9% with a mean of 8.1%. Additionally, the rate of PPH for both PD and DP varied from less than 1 up to 32% with a mean of 8.1% and from 0 up to 8.6% with a mean of 3.5%, respectively.

### RCTs impacting the rate of PPH

Among 99 RCTs, five^28,30,34,98,103^ reported a statistically significant reduction of PPH (Table 5), all after PD. No RCT reported a statistically significant impact on the rate of PPH after DP. One RCT focused on pancreaticojejunostomy (PJ) technique^28^, one focused on a falciform ligament wrap around the hepatic artery^30^, one focused on the comparison between PJ and pancreaticogastrostomy (PG) techniques^34^, and two RCTs focused on perioperative interventions^98,103^.

**Table 5:** Statistically significant RCTs.

Year	Authors	Primary aim	PPH results	Total PPH rate (%)
2020	Di Mola *et al*.^28^	End-to-side duct-to-mucosa PJ after PD. A comparison trial of small versus large jejunal incision	PPH [small 2 (8%) vs large 8 (36%); *P*=0.018]	PPH: 21 PPH A: 10.4 PPH B: 8.3 PPH C: 2
2021	Welsch *et al*.^30^	To investigate whether a prophylactic falciform ligament wrap around the hepatic and gastroduodenal artery can prevent PPH from these vessels.	mITT analysis: PPH from HA/GDA mITT analysis [wrap 6 (2.9%) vs no wrap 15 (7.1%), *P*=0.071] PPH B/C [wrap 20 (9.7%) vs no wrap 31 (14.8%), *P*=0.13] PPH B/C events per patients [wrap 24 (11.6%) vs no wrap 42 (20%), *P*=0.041] PP analyses: PPH from HA/GDA PP analysis [wrap 4(2%) vs no wrap 15 (7.2%), *P*=0.017] PPH B/C [wrap 18 (9%) vs no wrap 31 (15%), *P*=0.06] PPH B/C events per patients [wrap 21 (10.5%) vs no wrap 42 (20.3%), *P*=0.017]	PPH (mITT): 12.2 PPH (PP): 11.7
2016	Keck *et al*.^34^	To assess pancreatic fistula rate and secondary endpoints after PG versus PJ in PD	PPH [PG 17 (11%) vs PJ 36 (21%), *P*=0.023] PPH A [PG 9 (5%) vs PJ 1 (1%); no *P*-value] PPH B [PG 16 (9%) vs PJ 6 (4%); *P*-value] PPH C [PG 11 (6%) vs PJ 10 (7%); no *P*-value]	PPH: 17 PPH A: 3 PPH B: 7 PPH C: 7
2020	Tarvainen *et al*.^103^	To assess the noninferiority of hydrocortisone compared with pasireotide in reducing complications after partial pancreatectomy	PPH [Pasireotide 0 (0%) vs Hydrocortisone 7 (11%), *P*=0.01] PPH B/C [Pasireotide 0 (0%) vs Hydrocortisone 6 (10%), *P*=0.01] Any PPH (PD) [Pasireotide 0 (0%) vs Hydrocortisone 4 (15%), *P*=0.044; PPH B/C (PD) [Pasireotide 0 (0%) vs Hydrocortisone 4 (15%), *P*=0.044] Any PPH (DP) [Pasireotide 0 (0%) vs Hydrocortisone 3 (10%), *P*=0.23]; PPH B/C (DP) [Pasireotide 0 (0%) vs Hydrocortisone 2 (7%), *P*=0.49).	PPH 5.5 PPH A: 0.7 PPH B/C: 4.7
2022	Smits *et al*.^98^	To design a multimodal algorithm for the early recognition and minimally-invasive management of postoperative complications in patients having pancreatic resection for all indications	PPH requiring intervention [PORSCH 47(5%) vs standard 51(6%), *P*=0.046]	PPH: 5.6

ETS, externalized trans-anastomotic stents; GDA, gastroduodenal artery; HA, hepatic artery; mITT, modified intention-to-treat; PD, pancreatoduodenectomy; PG, pancreaticogastrostomy; PJ, pancreaticojejunostomy; PP, per-protocol; PPH, postpancreatectomy hemorrhage.

First, a 2020 Italian monocenter RCT^28^ in 48 patients undergoing PD compared a small (incision as large as the diameter of the pancreatic duct) versus larger jejunal incision (incision as large as the upper-lower extent of the pancreatic remnant) when performing end-to-side duct-to-mucosa PJ. The study found a decreased rate of PPH when performing a small jejunal incision (PPH 8 vs 36%; *P*=0.018). Additionally, the multivariate analysis confirmed large jejunal incision, together with delayed gastric emptying, to be independent predictors for PPH (large jejunal incision anastomosis: Odds ratio (OR)=12.71, 95% CI: 1.23–131.55, *P*= 0.033).

Second, a 2016 German multicenter RCT^30^ in 445 patients assessed whether a prophylactic falciform ligament wrap around the gastroduodenal artery stump and hepatic artery can prevent PPH. Among 417 patients included in the per-protocol (PP) analysis, a statistically significant reduction [OR 0.26 (95% C.I.: 0.09–0.80), *P*=0.017 (Fisher’s test)] of the primary endpoint (PPH from hepatic artery/gastroduodenal artery) was reported.

Third, another 2016 multicenter German RCTs^34^ in 440 patients undergoing PD compared the rate of POPF after PG versus PJ. As secondary outcome the study reported a statistically significant reduced rate of PPH grades A–C in the PJ group (11 vs 21%, *P*=0.023).

Fourth, a 2020 Finnish monocenter RCT^103^ in 281 patients assessed the noninferiority of hydrocortisone compared with pasireotide in reducing complications after partial pancreatectomy. The rate of PPH was assessed as secondary outcome and was reduced in the pasireotide group [Any PPH: 0 (0%) vs 7 (11%), *P*=0.01; PPH B/C 0 (0%) vs 6 (10%), *P*=0.01]. Additionally, a sub-analysis reported that the difference in PPH rate between pasireotide and hydrocortisone was statistically significant only in patients undergoing PD [Any PPH: 0 (0%) vs 4 (15%), *P*=0.044; PPH B/C 0 (0%) vs 4 (15%), *P*=0.044] and not in those undergoing DP [Any PPH: 0 (0%) vs 3 (10%), *P*=0.23; PPH B/C 0 (0%) vs 2 (7%), *P*=0.49].

Fifth, a 2022 Dutch Pancreatic Cancer Group open-label, nationwide, stepped-wedge cluster-randomized RCT (PORSCH trial)^98^, implemented a multimodal algorithm for the early recognition and minimally-invasive management of postoperative complications in 1748 patients having pancreatic resection for all indications. The primary outcome was a composite including PPH that required invasive intervention, new-onset organ failure, and death either during admission or within 90 days after resection (the outcome was met if any of these events occurred). The primary outcome occurred in 8% (73/863) of patients in the intervention group and in 14% (124/885) of patients in the control group (adjusted Risk Ratio 0.48, 95% CI: 0.38–0.61; *P*<0.0001). In the secondary outcomes, PPH requiring intervention occurred in 47 patients (5%) in the intervention group versus 51 patients (6%) in the control group (adjusted RR 0.65, 95% CI: 0.42–0.99; *P*=0.046).

## Discussion

This systematic review including RCTs which reported on the prevention and mitigation of PPH found an incidence rate of 8.8% for PPH grade B/C. After assessing 99 RCTs, five reported an approach which reduced the rate of PPH. Three of these concerned surgical technique: a small jejunal incision when performing an end-to-side duct-to-mucosa PJ^28^, a falciform ligament patch around hepatic artery and stump of gastroduodenal artery during PD^30^, and performing PJ instead of PG^34^. Two concerned perioperative patient management: administration of perioperative pasireotide instead of hydrocortisone^103^, and a multimodal algorithm for the postoperative management of pancreatic patients^98^.

All 99 studies included in the present systematic review are RCTs providing a high-level of evidence. Among all RCTs in the present systematic review, only two focused specifically on the treatment, prevention, and management of PPH as their primary aim. This highlights the fact that more RCTs focusing on PPH are needed. Interestingly, none of the RCTs, including the five RCTs which reported a reduction of PPH, reported a reduction of POPF. These data suggest that even though PPH is strongly associated with POPF, there must be other independent factors which affect the PPH incidence. However, an alternative explanation could be that the association between POPF and PPH is so strong that even a small nonsignificant reduction of POPF could result in a reduction of a rare event such as PPH.

PPH poses a complex challenge as it consistently provides an emergency situation. Scientific research has primarily focused on preventing and detecting PPH early, while investigating its management through RCTs remains difficult due to ethical controversies of asking for patient consent in an emergency situation. Consequently, research efforts primarily focus on technical aspects such as arterial stump closure, intraoperative hemostasis, and early radiological prevention and diagnosis of PPH. In this regard, radiology, specifically CT scans, plays a pivotal role in the diagnostic algorithm for PPH. CT scans offer the advantage of being readily available, providing quick access, and exhibiting high sensitivity in identifying bleeding.

Strategies to mitigate the severity of PPH could decrease postoperative mortality and associated length of hospital stay. A 2020 Italian single center RCT^36^ in 72 patients compared PJ with an externalized trans-anastomotic stent versus PG with externalized stent in patients with a high-risk pancreatic anastomosis. The incidence of PPH in both groups did not differ significantly (9 vs 14%, *P*=0.31). However, the study did report a statistically significant (*P*=0.046) difference, albeit somewhat conflicting, distribution of PPH severity grades between the PG group and PJ group (13.9 vs 0%) with grade A, (13.9 vs 22.2%) with grade B, and (11.1 vs 2.8%) with grade C.

How should the results of the present systematic review impact our clinical patient management? On the one hand, all statistically significant RCTs deal with different approaches, which are not mutually exclusive and allow us to apply them simultaneously. This scenario gives us multiple tools to prevent PPH, potentially reducing morbidity and mortality. On the other hand, the absence of confirmatory studies remains an uncertainty. Furthermore, it is important to highlight that the five RCTs have widely varying sample sizes, ranging from 48 to 1748 patients and were all performed in medium to high-volume centers performing over 20 pancreatectomies per year. The latter aspect is important for the external validity of study results as high-volume centers typically have well-functioning multidisciplinary teams with experienced interventional endoscopists and interventional radiology.

The present review reveals a significant disparity in the incidence of PPH between PD and DP, with PPH occurring nearly 2.5 times more frequently in PD compared to DP (8.1 versus 3.5%). These findings underscore the importance of considering the gastroduodenal and hepatic arteries as potential sources of PPH. Notably, the only RCT specifically targeting the prevention of PPH from these arteries reported noteworthy and promising results^30^.

While the reduction in PPH rate following DP is evident, complete resolution remains elusive. This persistent issue can be attributed to the influence of additional contributing factors in PPH development. Bleeding from the resection margin after DP has consistently been a concern. Despite numerous RCTs attempting to mitigate this complication through various techniques such as patches, glues and stapling, none have succeeded in substantially reducing the overall incidence of PPH suggesting the possible involvement of unclear factors.

Finally, in the present systematic review several RCTs used other definitions for PPH than the ISGPS definition. Although this could influence the incidence of PPH it will not impact the effectiveness much as both arms of the RCTs use the same definitions. Indeed, the ISGPS classification of PPH, albeit being the global standard, does not give any treatment advice. A uniform guidance on the management of PPH would be useful but would require more high-quality studies as these are currently scarce. Indeed, among PPH grade B both early and late hemorrhage are considered despite having a different etiology and therefore different management and treatment, particularly now that endovascular treatment is the preferred approach^114^.

Over the past decade, thanks to advances in in predictive modeling and artificial intelligence, the prospect of preventing or mitigating life-threatening complications has improved. The scientific community must prioritize high-quality research in critical new areas such as radiomics, where current knowledge is lacking. This emphasis is essential to definitively enhance our ability to prevent and manage postoperative complications, including PPH.

Finally, over time PPH has evolved to be defined, as POPF, a multifactorial condition, emphasizing the need for a multidisciplinary approach even in the scientific research setting leading the way to a key role for clinical translational medicine in the future.

Some limitations should be taken into account when interpreting these results. First, the present systematic review describes a period of 11 years in which several surgical and medical improvements have been achieved. Second, PPH was always analyzed as a secondary endpoint, therefore it is not possible to ascertain whether the included RCTs were adequately powered to detect differences in PPH incidence or severity. Third, the included RCTs did not take specific risk groups into account. Indeed, it is well-established that patients with specific anatomical and surgical characteristics (e.g. small pancreatic duct^115–117^, high acinar count >60%^118^, obesity^116^) have a higher risk of developing POPF and related postoperative complications. Fourth, the limited number of international RCTs may affect the generalizability of the present findings, as these may be influenced by specific regional or institutional treatment factors. The main strength of this study is its systematic approach including only RCTs, thus providing the highest possible level of evidence.

## Conclusions

In conclusion, this first systematic review identified five strategies for the prevention of PPH, which should be further investigated within high-quality international multicenter RCTs. RCTs focusing on the management of PPH are lacking and further multicenter studies are encouraged in this field.

## Ethical approval

None.

## Consent

None.

## Sources of funding

None.

## Author contribution

R.M.M. and B.Z.: drafted the manuscript; R.M.M. and M.G.B.: develop the search strategy; M.G.B., G.M., and G.M.: provided statistical expertise; M.G.B., G.K., and R.S.: provided expertise on postpancreatectomy hemorrhage. All authors contributed in read, provided feedback, and approved the final manuscript. All authors contributed to the development of the selection criteria, the risk of bias assessment strategy, and data extraction criteria.

## Conflicts of interest disclosure

The authors declare that they have no financial conflict of interest with regard to the content of this report.

## Research registration unique identifying number (UIN)

Systematic review registered on Prospero registry. Unique Identifying Number (UIN): CRD42023409666.

## Guarantor

Marc G. Besselink.

## Data availability statement

The data that have been used are available at the Evidence Map of Pancreatic Surgery website (https://www.evidencemap.surgery).

## Provenance and peer review

Not commissioned, externally peer-reviewed.

## Supplementary Material

SUPPLEMENTARY MATERIAL
