# Supplementary material for: Treatment strategies to prevent or mitigate the outcome of postpancreatectomy hemorrhage: a review of randomized trials
Source: Int J Surg. 2023 Nov 16;110(10):6145–54. doi: 10.1097/JS9.0000000000000876 (PMC11486935; doi:10.1097/JS9.0000000000000876)
Supplement: SUPPLEMENTARY MATERIAL [file js9-110-6145-s003.docx]

## **Supplementary table 1. Included randomized clinical trials.**

| **Evidence Map Section** | **Year** | **Authors** | **Study design** | **Surgery** | **Topic** | **Sample size** |
| --- | --- | --- | --- | --- | --- | --- |
| Pancreatic anastomosis:  Stenting | 2017 | Bin et al. | National multicenter | PD | Stenting vs no stenting | 87 |
|  | 2018 | Qureshi et al. | Monocentric | PD | Stenting vs no stenting | 102 |
|  | 2021 | Singh et al. | Monocentric | PD | Stenting vs no stenting | 50 |
|  | 2016 | Jang et al. | National multicenter | PD | Internal vs external stenting | 328 |
|  | 2019 | Shin et al. | Monocentric | PD | Internal vs external stenting | 213 |
| Pancreatic anastomosis:  Technique | 2015 | Xu et al. | Monocentric | PD | Duct-to-mucosa vs papillary-like main pancreatic duct invaginated PJ | 308 |
|  | 2015 | El Nakeeb et al. | Monocentric | PD | Duct-to-mucosa vs invagination | 107 |
|  | 2016 | Bai et al. | Monocentric | PD | Duct-to-mucosa vs invagination | 132 |
|  | 2018 | Singh et al. | Monocentric | PD | Duct-to-mucosa vs dunking | 193 |
|  | 2018 | Senda et al. | Monocentric | PD | Duct-to-mucosa vs invagination | 120 |
|  | 2019 | Hirono et al. | Monocentric | PD | Modified Blumgart Mattress Suture vs Conventional Interrupted Suture | 224 |
|  | 2013 | Qin et al. | Monocentric | PD | Pancreas invagination and suturing vs jejunal mucosa cauterization and pancreas sutured with jejunal muscle layer | 46 |
|  | 2020 | Di Mola et al. | Monocentric | PD | Small jejunal incision vs large jejunal incision | 48 |
| Pancreatic anastomosis:  Additional interventions | 2021 | Tangtawee et al. | Monocentric | PD | Omental roll- up PJ vs non-omental roll- up PJ | 68 |
|  | 2021 | Welsch et al. | National multicenter | PD | Falciform ligament wrap PJ vs non falciform ligament wrap PJ | 445 |
| Pancreatic anastomosis:  PG vs PJ | 2012 | Wellner et al. | Monocentric | PD | PD with PG Vs PD with PJ | 116 |
|  | 2013 | Topal et al. | National multicenter | PD | PD with PG Vs PD with PJ | 167 |
|  | 2013 | Figueras et al. | National multicenter | PD | PD with PG Vs PD with PJ | 123 |
|  | 2016 | Keck et al. | National multicenter | PD | PD with PG Vs PD with PJ | 440 |
|  | 2020 | Eguchi et al. | Monocentric | PD | PD with PG Vs PD with PJ | 60 |
|  | 2020 | Andrianello et al. | Monocentric | PD | PD with PG Vs PD with PJ | 72 |
| Entero-enteric anastomosis:  Pylorus-preserving vs pylorus resecting | 2014 | Matsumoto et al. | Monocentric | PD | PPPD vs SSPPD | 100 |
|  | 2018 | Hackert et al. | Monocentric | PD | Pylorus-preserving PD vs Pylorus-Resecting PD | 188 |
| Entero-enteric anastomosis:  Pylorus-preserving vs Whipple procedure | 2022 | Busquets et al. | Monocentric | PD | Pylorus Preserving PD vs Standard Whipple's Procedure | 84 |
| Entero-enteric anastomosis:  antecolic vs retrocolic | 2014 | Tamandl et al. | Monocentric | PD | Antecolic vs retrocolic | 64 |
|  | 2014 | Imamura et al. | Monocentric | PD | Antecolic vs retrocolic | 116 |
|  | 2014 | Eshuis et al. | National multicenter | PD | Antecolic vs retrocolic | 246 |
|  | 2020 | Toyama et al. | National multicenter | PD | Antecolic vs retrocolic | 214 |
| Entero-enteric anastomosis:  Billroth II vs Roux-En-Y | 2019 | Busquets et al. | Monocentric | PD | Billroth II vs Roux-En-Y | 80 |
| Entero-enteric anastomosis:  Braun entero-enterostomy | 2014 | Wang et al. | Monocentric | PD | Modified BEE vs traditional gastrojejunostomy | 62 |
|  | 2015 | Kakaei et al. | Monocentric | PD | Braun vs No Braun | 30 |
|  | 2016 | Hwang et al. | Monocentric | PD | Braun vs No Braun | 60 |
| Entero-enteric anastomosis:  Other anastomotic technique | 2016 | Sakamoto et al. | National multicenter | PD | Circular stapler vs hand-sewn | 101 |
| Drainage:  abdominal drainage vs no drainage | 2014 | Van Buren et al. | National multicenter | PD | Drain vs no drain | 137 |
|  | 2016 | Witzigmann et al. | National multicenter | PD | Drain vs no drain | 438 |
| Drainage:  Early vs late removal of drainage | 2019 | Dembinski et al. | National multicenter | PD | Early vs late | 141 |
|  | 2020 | Dai et al. | Monocentric | PD | Early vs late | 144 |
|  | 2021 | Dai et al. | National multicenter | PD | Early vs late | 312 |
| Drainage:  Type of drainage | 2018 | Cecka et al. | National multicenter | PD | Closed-suction drains vs passive gravity drains | 223 |
| Surgical aspects:  Extended vs standard resection | 2014 | Jang et al. | National multicenter | PD | Extended vs standard resection | 244 |
|  | 2016 | Sperling et al. | Monocentric | PD | Extended vs standard resection | 103 |
|  | 2017 | Ignjatovic et al. | Monocentric | PD | Extended vs standard resection | 60 |
|  | 2021 | Wang et al. | Monocentric | PD | Extended vs standard resection | 240 |
| Surgical aspects:Surgical approach | 2019 | Sabater et al. | National multicenter | PD | Standard approach vs Artery-first approach | 176 |
| Surgical aspects: Isolated Roux-En-Y PJ | 2014 | Tani et al. | Monocentric | PD | Isolated Roux-en-Y vs conventional reconstruction | 153 |
|  | 2014 | El Nakeeb et al. | Monocentric | PD | Isolated Roux PJ vs Pancreaticogastrostomy | 90 |
| Surgical aspects:  Energy device in dissection | 2012 | Uzunoglu et al. | International Multicenter | PD | Ultrasonic vs conventional | 101 |
|  | 2013 | Uzunoglu et al. | Monocentric | PD | LigaSure vs Conventional | 136 |
|  | 2020 | Gehrig et al. | Monocentric | PD | LigaSure vs Conventional | 171 |
| MIPD:  Minimally-invasive vs Open PD | 2017 | Palanivelu et al. | Monocentric | PD | Laparoscopic PD vs Open PD | 64 |
|  | 2018 | Poves et al. | National multicenter | PD | Laparoscopic PD vs Open PD | 66 |
|  | 2019 | Van Hilst et al. | National multicenter | PD | Laparoscopic PD vs Open PD | 38 |
|  | 2019 | Van Hilst et al. | National multicenter | PD | Laparoscopic PD vs Open PD | 42 |
|  | m2021 | Wang et al. | National multicenter | PD | Laparoscopic PD vs Open PD | 656 |
| Pancreatic remnant:  Reinforced staplers | 2012 | Hamilton et al. | Monocentric | DP | Mesh reinforcement vs no-mesh reinforcement | 100 |
|  | 2019 | Kondo et al. | National multicenter | DP | Reinforced stapler vs bare stapler | 122 |
|  | 2021 | Wennerblom et al. | National multicenter | DP | Reinforced stapling vs standard stapling | 107 |
| Pancreatic remnant:  Anastomosis | 2014 | Antila et al. | Monocentric | DP | Roux-Y Binding PJ vs hand-sewn | 47 |
|  | 2016 | Kawai et al. | National multicenter | DP | PJ vs stapling closure | 123 |
|  | 2017 | Uemura et al. | National multicenter | DP | Duct-to-mucosa PG vs hand-sewn closure | 80 |
| Pancreatic remnant:  Autologous coverage | 2016 | Hassenpflug et al. | National multicenter | DP | Teres ligament patch vs no teres ligament patch | 152 |
| Pancreatic remnant:  Sealant | 2012 | Montorsi et al. | National multicenter | DP | Tachosil vs no Tachosil | 275 |
|  | 2015 | Sa Cunha et al. | National multicenter | DP | Tachosil vs no Tachosil | 270 |
|  | 2016 | Park et al. | National multicenter | DP | Tachosil vs no Tachosil | 101 |
|  | 2021 | Mungroop et al. | National multicenter | DP | Tachosil vs no Tachosil | 247 |
| Drainage:  Drain Vs No drain | 2017 | Van Buren et al. | National multicenter | DP | Drain vs no drain | 344 |
| Drainage:  Early removal Vs late removal | 2020 | Dai et al. | Monocentric | DP | Early vs late | 144 |
| Drainage:  Type of drainage | 2018 | Cecka et al. | National multicenter | DP | Passive drain vs closed-suction drain | 223 |
| Surgical aspects:  Spleen management | 2021 | Yamada et al. | National multicenter | DP | Separate division vs combined division | 318 |
| Surgical aspects:  Energy device dissection | 2021 | Landoni et al. | National multicenter | DP | Stapled vs ultrasonic transection | 145 |
| MIDP:  Minimally-invasive Vs Open DP | 2019 | De Rooij et al. | National multicenter | DP | MIDP vs ODP | 111 |
|  | 2020 | Bjornsson et al. | Monocentric | DP | LDP vs ODP | 60 |
| Various perioperative interventions:  Prevention of surgical site infections | 2018 | Yamamoto et al. | National multicenter | PD | 1-day antimicrobial prophylaxis vs 5-days prophylaxis | 82 |
|  | 2021e | Andrianello et al. | Monocentric | PD | PICO vs standard sterile dressing | 100 |
|  | 2020 | Singh et al. | Monocentric | PD | Bile clamping vs no bile clamping | 40 |
|  | 2020 | De Pastena et al. | Monocentric | PD | Wound protector vs standard drape | 212 |
| Various perioperative interventions:  Interventions to improve recovery | 2017 | Deng et al. | Monocentric | PD | Modified ERAS vs no ERAS | 159 |
|  | 2019 | Hwang et al. | Monocentric | PD | ERAS vs no ERAS | 276 |
|  | 2019 | Takagi et al. | Monocentric | PD | ERAS vs no ERAS | 80 |
|  | 2021 | Ergenc et al. | Monocentric | PD, DP, other | ERAS vs no ERAS | 38 |
| Various perioperative interventions:Perfusion management | 2015 | Van Samkar et al. | International multicenter | PD | Crystalloid fluid restriction vs standard fluid therapy | 66 |
|  | 2017 | Weinberg et al. | National multicenter | PD | Goal directed therapy vs standard fluid therapy | 52 |
| Various perioperative interventions:Other intervention to improve outcomes | 2016 | Zhang et al. | Monocentric | PD | Preoperative Ulinastatin vs no Ulinastatin | 106 |
|  | 2020 | Bergeat et al. | Monocentric | PD | Nasogastric tube up to 3-5 days vs no nasogastric tube | 111 |
|  | 2021 | Smith et al. | Monocentric | PD, DP | Intraoperative secretin vs standard therapy | 170 |
|  | 2022 | Jeong et al. | Monocentric | PD, DP, TP | Preemptive albumin administration vs standard therapy | 200 |
|  | 2022 | Smits et al. | National multicenter | PD, DP, other | PORSCH vs Standard | 1748 |
| Pharmaceutical co-treatment:  Somatostatin analogues | 2013 | Belyaev et al. | Monocentric | DP | Intraoperative intra-arterial octreotide vs no octreotide | 25 |
|  | 2018 | El Nakeeb et al. | Monocentric | PD | Postoperative octreotide vs standard | 104 |
|  | 2021 | Cao et al. | National multicenter | PD | Prophylactic somatostatin vs no somatostatin | 205 |
| Pharmaceutical co-treatment:  corticosteroids | 2016 | Laaninen et al. | Monocentric | PD | Perioperative hydrocortisone vs placebo | 155 |
|  | 2020 | Tarvainen et al. | Monocentric | PD, DP, other | Pasireotide vs Hydrocortisone | 126 |
| Nutrition:  Enrich Vs Standard diet | 2013 | Zhu et al. | Monocentric | PD | Polyunsaturated fatty acid diet vs no polyunsaturated fatty acid diet | 76 |
|  | 2014 | Aida et al | Monocentric | PD | Preoperative oral immunonutrition vs standard nutrition |  |
| Nutrition:  Route of nutrition | 2012 | Park et al. | Monocentric | PD | EN vs TPN | 40 |
|  | 2016 | Perinel et al. | National multicenter | PD | EN vs TPN | 204 |
|  | 2019 | Wu et al. | National multicenter | PD | Enteral feeding vs oral feeding | 114 |
| Nutrition:  Time point and duration of nutritional support | 2021 | Liu et al. | Monocentric | PD | Early nasojejunal feeding vs early oral feeding vs saline | 120 |
| Other surgical aspect:  Treatment of complications | 2015 | Fujii et al. | National multicenter | PD | Dietary intake vs no dietary intake | 59 |
|  | 2015 | Fujii et al. | National multicenter | DP | Dietary intake vs no dietary intake | 30 |
| Other surgical aspect:Parenchyma-sparing interventions | 2017 | Chen et al. | Monocentric | Other | Robot-assisted middle pancreatectomy vs open MP | 137 |
| Other surgical aspect:Total vs PD | 2022 | Balzano et al. | National multicenter | TP, PD | Total pancreatectomy + IAT vs Pancreatoduodenectomy | 61 |

*PD: pancreatoduodenectomy; DP: distal pancreatectomy; MP: middle pancreatectomy; TP: total pancreatectomy; PJ: pancreaticojejunostomy; PG: pancreaticogastrostomy; PPPD: pylorus-preserving pancreatoduodenectomy; SSPPD: subtotal stomach-preserving pancreatoduodenectomy; BEE: Braun entero-enterostomy; MIDP: minimally-invasive distal pancreatectomy; LDP: laparoscopic distal pancreatectomy; ODP: open distal pancreatectomy; NPWT: negative pressure wound therapy; ERAS: enhanced recovery after surgery; EN: enteral nutrition; TPN: total parenteral nutrition; IAT: islet autotranplantation;*
