# Supplementary material for: Treatment strategies to prevent or mitigate the outcome of postpancreatectomy hemorrhage: a review of randomized trials
Source: Int J Surg. 2023 Nov 16;110(10):6145–54. doi: 10.1097/JS9.0000000000000876 (PMC11486935; doi:10.1097/JS9.0000000000000876)
Supplement: SUPPLEMENTARY MATERIAL [file js9-110-6145-s004.docx]

## **Supplementary table 2. Results of RCTs on pancreatoduodenectomy**

| **Year** | **Authors** | **Topic** | **Primary aim** | **PPH Results** | **Total PPH  rate (%)** | **Mortality  PPH related (%)** |
| --- | --- | --- | --- | --- | --- | --- |
| 2017 | Bin et al. | Stenting vs no stenting | Evaluate the clinical effect of different PJ techniques in the treatment of PD and investigate the applicability of PJ without pancreatic duct stenting | PPH (stent 1 [2.3%] vs no stent 2 [4.5%]; p= 0.57) | 3.4 | 0 |
| 2018 | Qureshi et al. | Stenting vs no stenting | Compare POPF rates in patients with stented versus non-stented PJ | Bleeding gastrojejunostomy (stent 4 [7.5%] vs no stent 2 [4%]; p= 0.46)  Bleeding in patient with CR-POPF (stent 0 vs no stent 0) | - | - |
| 2021 | Singh et al. | Stenting vs no stenting | Internal stenting of PJ to reduce POPF PD | PPH (stent 1 [4%] vs no stent 1 [4%]; p= 0.6) | 4 | 0 |
| 2016 | Jang et al. | Internal vs external stenting | To investigated the effect of two ways of pancreatic stenting after PD on the rate of POPF. | Late PPH (external 7 [4.3%] vs internal 3 [1.8%] p=0.33)  Early PPH (external 4 [2.4%] vs internal 1 [0.6%] p=0.21)  GI bleeding (external 2 [1.2%] vs internal 0 [0%] p=0.49) | 5.1 | 0 |
| 2018 | Shin et al. | Internal vs external stenting | To determine the most appropriate pancreatic drainage method, by investigating differences in patients implanted with external and internal pancreatic stents. | Late PPH (external 5 [5.2%] vs internal 3 [3.4%] p=0.56)  Early PPH (external 3 [3.1%] vs internal 1 [1.1%] p=0.62)  GI bleeding (external 2 [2.1%] vs internal 0 [0%] p=0.49) | 7.5 | 0 |
| 2015 | Xu et al. | Duct-to-mucosa vs papillary-like main pancreatic duct invaginated PJ | to reassess whether the new PJ would decrease the POPF rate. | PPH (Duct-to-mucosa n=2 vs invagination n=1, p=0.62) | 0.6 | 100 |
| 2015 | El Nakeeb et al. | Duct-to-mucosa vs invagination | To assess the surgical outcomes of duct to mucosa PJ (G1) and invagination PJ (G2) after PD | Internal hemorrhage (Duct-to-mucosa 3 [5.7%] vs invagination 2 [3.7%], p=0.68) | - | - |
| 2016 | Bai et al. | Duct-to-mucosa vs invagination | Comparison between invagination and duct-to-mucosa PJ techniques in preventing POPF and other AEs. | PPH (Duct-to-mucosa 2 [3.1%] vs invagination 4 [5.9%], p=0.68) | 4.5 | 100 |
| 2018 | Singh et al. | Duct-to-mucosa vs dunking | To compare the two most common techniques of PJ (duct-to-mucosa and dunking) in a randomized trial | PPH (Duct-to-mucosa 5 [5.2%] vs dunking 5 [5.2%], p=0.99) | 5.1 | - |
| 2019 | Hirono et al. | Modified Blumgart Mattress Suture vs Conventional Interrupted Suture | To evaluate whether mattress suture of pancreatic parenchyma and the seromuscular layer of jejunum (modified Blumgart method) during PJ decreases the incidence of clinically relevant POPF after PD | PPH (Duct-to-mucosa 0 [0] vs mattress suture 1 [0.9%], p=0.32) | 0.48 | 0 |
| 2013 | Quin et al. | Pancreas invagination and connecting part was suturing vs jejunal mucosa cauterization, pancreas sutured with jejunal muscle layer and jejunum and pancreas were sutured | To compare the retention or the removal of the jejunal mucosa during pancreatic duct to jejunal anastomosis after PD | Delayed remnant pancreatic bleeding (1 retention [4.3%] vs removal 1 [4.3%], p= 1) | 6.9 | 0 |
| 2020 | Di Mola et al. | **S**mall vs large jejunal incision | End-to-side duct-to-mucosa PJ after PD. A comparison trial of small versus larger jejunal incision | PPH (small 2 [8%] vs large 8 [36%]; p= 0.018) | PPH: 21  PPH A: 10.4  PPH B: 8.3  PPH C: 2 | - |
| 2021 | Tangtawee et al. | **O**mental roll- up PJ vs non-omental roll- up PJ | To compare outcomes between omental roll- up and non- omental roll- up PJ anastomosis | PPH (omental roll-up 1 [2.9%] vs non-omental roll-up 1 [2.9%], p=1) | 2.9 | - |
| 2021 | Welsch et al. | **F**alciform ligament wrap PJ vs non falciform ligament wrap PJ | To investigate whether a prophylactic falciform ligament wrap around the hepatic and gastroduodenal artery can prevent PPH from these vessels. | mITT analysis:  PPH from HA/GDA mITT analysis (wrap 6 [2.9%] vs no wrap 15 [7.1%], p= 0.071)  PPH B/C (wrap 20 [9.7%] vs no wrap 31 [14.8%], p= 0.13) PPH B/C events per patients (wrap 24 [11.6%] vs no wrap 42 [20%], p= 0.041) PP analyses: PPH from HA/GDA PP analysis (wrap 4[2%] vs no wrap 15 [7.2%], p= 0.017)  PPH B/C (wrap 18 [9%] vs no wrap 31 [15%], p= 0.06)  PPH B/C events per patients (wrap 21 [10.5%] vs no wrap 42 [20.3%], p= 0.017) | mITT: 12.2  PP:11.7 | - |
| 2012 | Wellner et al. | PD with PG Vs PD with PJ | To compare the perioperative outcome of PD with PG vs PJ | PPH (PG 6 [10%] vs PJ 4 [7%], p= 0.73)PPH B/C (PG 2 [3%] vs PJ 1 [2%], p= 1.00)PPH Extra-luminal (PG 1 [2%] vs PJ 3 [5%], p= 0.36)PPH Intra-luminal (PG 4 [7%] vs 1 [2%], p= 0.36) | 9 | - |
| 2013 | Topal et al. | PD with PG Vs PD with PJ | To compare the outcomes of different reconstructive techniques in patients undergoing PD for pancreatic or periampullary tumors. | Hemorrhage (PG 21 [13%] vs PJ 17 [10%], p= 0.49) | 11.5 | - |
| 2013 | Figueras et al. | PD with PG Vs PD with PJ | Comparing PG with PJ after PD was to test the hypothesis that invaginated PG would result in a lower rate and severity of pancreatic fistula | PPH (PG 13 [20%] vs PJ 7 [12%], p= 0.23)PPH A (PG 2 [3%] vs PJ 0 [0%])PPH B (PG 2 [3%] vs PJ 2 [3%])PPH C (PG 9 [14%] vs PJ 5 [9%]) | PPH: 16.2PPH A: 1.6PPH B: 3.25PPH C: 11.3 | - |
| 2016 | Keck et al. | PD with PG Vs PD with PJ | To assess pancreatic fistula rate and secondary endpoints after PG versus PJ in PD | PPH A (PG 9 [5%] vs PJ 1 [1%]; no p-value)PPH B (PG 16 [9%] vs PJ 6 [4%]; p= 0.02)PPH C (PG 11 [6%] vs PJ 10 [7%]; no p-value) | PPH: 17PPH A: 3PPH B: 7PPH C: 7 | - |
| 2020 | Eguchi et al. | PD with PG Vs PD with PJ | To compare PG and PJ with regard to the incidence of DGE after PD | Intra-abdominal hemorrhage (PG 2/25 [7/93] vs PJ 2/24 [8/92], p= 0.99) | 7.5 | 0 |
| 2020 | Andrianello et al. | PD with PG Vs PD with PJ | To investigate whether PJ or PG, both with externalized transanastomotic stent, is the best reconstruction method for patients at high risk of POPF after PD | PPH (PG with ETS 14 [38.9%] vs PJ with ETS 9 [25.0%]; p= 0.31)PPH A (PG with ETS 5 [13.9%] vs PJ with ETS 0 [0%])PPH B (PG with ETS 5 [13.9%] vs PJ with ETS 8 [22.2%]) PPH C (PG with ETS 4 [11.1%] vs PJ with ETS 1 [2.8%])  Different distribution of PPH grades (PG group: 5 [13.9%] grade A, 5 [13.9%] grade B, and 4 [11.1%] grade C vs PJ group: 0 grade A, 8 [22.2%] grade B, and 1 [2.8%] grade C; p = .046) | PPH: 31.9 PPH A: 6.9  PPH B: 18  PPH C: 6.9 | - |
| 2014 | Matsumoto et al. | PPPD vs SSPPD | To compare perioperative complications and long‐term nutritional status with pylorus-preserving PD and subtotal stomach-preserving PD | PPH C (PPPD 1 [2%] vs SSPPD 1 [2%], p= 1.0) | PPH C: 2 | 0 |
| 2018 | Hackert et al. | Pylorus-preserving PD vs Pylorus-Resecting PD | To investigate the effect of pylorus resection on postoperative DGE after partial PD | Postoperative hemorrhage (PP 9 [9.5%] vs PR 9 [9.7%], p= 0.96) | 9.5 | - |
| 2022 | Busquets et al. | Pylorus Preserving PD vs Standard Whipple's Procedure | To compare the incidence of DGE among PD techniques. | Postoperative hemorrhage (PP 2 [4.8%] vs Whipple 6 [14.3], p= 0.137) | 10.1 | 40 |
| 2014 | Tamandl et al. | Antecolic vs retrocolic | To determine if performing duodenojejunostomy via the antecolic rather than the retrocolic route improved incidence of DGE | Bleeding (antecolic 2 [5.9%] vs retrocolic 1 [3.8%], p= 0.55) | 5 | - |
| 2014 | Imamura et al. | Antecolic vs retrocolic | To investigate the influence of the reconstruction route on post-operative gastric emptying and nutrition | PPH (antecolic 3 [5.2%] vs retrocolic 3 [5.2%], p= 1.0) | 5.2 | - |
| 2014 | Eshuis et al. | Antecolic vs retrocolic | To investigate the relationship between the route of gastroenteric (GE) reconstruction after PD and the postoperative incidence of delayed gastric emptying | PPH A (antecolic 1 [1%] vs retrocolic 1 [1%], p= 0.08)PPH B (antecolic 5 [4%] vs retrocolic 3 [2%], no p-value)PPH C (antecolic 1 [1%] vs retrocolic 9 [7%], no p-value) | PPH: 8.1 PPH A: 0.8  PPH B: 3.2 PPH C: 4 | - |
| 2020 | Toyama et al. | Antecolic vs retrocolic | To determine whether retrocolic reconstruction is non-inferior to antecolic reconstruction in terms of DGE incidence after PD and investigated patients’ postoperative nutritional status | PPH (antecolic 7 [6.8%] vs retrocolic 7 [6.5%], p= 0.37)PPH A (antecolic 2 [1.9%] vs retrocolic 0 [0%])PPH B (antecolic 3 [2.9%] vs retrocolic 4 [3.7%])PPH C (antecolic 2 [1.9%] vs retrocolic 3 [2.8%]) | PPH: 6.6 PPH A: 0.9  PPH B: 3.3 PPH C: 2.4 | - |
| 2019 | Busquets et al. | Billroth II vs Roux-En-Y | To compare Billroth II (single loop) and Roux-en-Y (double loop) after PD to determine whether Roux-en-Y reconstruction is associated with a lower incidence of DGE. | PPH (Billroth II 5 [13%] vs Roux-En-Y 3 [8%], p= 0.45) PPH B: (Billroth II 3 [8%] vs Roux-En-Y 3 [8%])  PPH C: (Billroth II 2 [5%] vs Roux-En-Y 0 [0%]) | PPH: 10 PPH B: 8 PPH C: 3 | - |
| 2014 | Wang et al. | Modified BEE vs traditional gastrojejunostomy | To compare outcomes of using a modified BEE (MBEE) with traditional gastrojejunostomy (TGJ), by inducting a purse-string suture instead of an additional anastomotic stoma. | Intrabdominal bleeding (TGJ 1[3.3%] vs MBEE 1[3.1%], p= 0.96)GI bleeding (TGJ 2 [6.6%] vs MBEE 5 [15.6%], p= 0.26) | 14 | 0 |
| 2015 | Kakaei et al. | Braun vs No Braun | To evaluate whether adding Braun jejunojejunostomy to a standard Whipple procedure would reduce postoperative complications. | Postop. Bleeding (no Braun 2[13.3%] vs Braun 1 [6.7%], p= 0.9) | 10 | - |
| 2016 | Hwang et al. | Braun vs no Braun | Investigates the clinical impact of Braun anastomosis on delayed gastric emptying (DGE) after pylorus-preserving PD (PPPD) | Bleeding (no Braun 1 [3.3%] vs Braun 0 [0%], p= 1) | 1.6 | 0 |
| 2016 | Sakamoto et al. | Circular stapler vs hand-sewn | To compare the incidence of DGE after circular stapler (CS) duodenojejunostomy with that of conventional hand-sewn (HS) anastomosis in PpPD | Intra-abdominal bleeding (CS n=0 vs HS n=1, p= 0.36)GI bleeding (CS n=0 vs HS n=0, not significant p-vale) | 1 | 0 |
| 2014 | Van Buren et al. | Drain vs no drain | To test by randomized prospective multicenter trial the hypothesis that PD without the use of intraperitoneal drainage does not increase the frequency or severity of complications | Intra-abdominal bleeding30 days (Drain 4 [6%] vs no drain 4 [6%], p= 1)60 days (Drain 4 [6%] vs no drain 6 [9%], p= 0.74)GI bleeding30 days (Drain 2 [3%] vs no drain 6 [9%], p= 0.27)60 days (Drain 2 [3%] vs no drain 7 [10%], p= 0.16) | 30 days: 11.660 days: 13.8 | 50 |
| 2016 | Witzigmann et al. | Drain vs no drain | To prove that omission of drains does not increase the reintervention rate after pancreatic head resection. | PPH (Drain 12 [6%] vs no drain 6 [3.1%], p= 0.17)PPH A (Drain 2 [1%] vs no drain 0 [0%])PPH B (Drain 0 [0%] vs no drain 1 [0.5%])PPH C (Drain 10 [5%] vs no drain 5 [2.6%]) | PPH: 4.6 PPH A: 0.5  PPH B: 0.3 PPH C: 3.8 | - |
| 2019 | Dembinski et al. | Early vs late | To determine whether the timing of removal of abdominal drainage after PD influences the 30-day surgical site infection (30-day SSI) rate | Postoperative hemorrhage (EDR 2 [2.9%] vs RDR 2 [2.8%], no p-value | 2.8 | - |
| 2020 | Dai et al. | Early vs late | To assess whether early drain removal after major pancreatectomy influences the incidence of complications in the patients with low risk of postoperative pancreatic fistula (POPF) | PPH (EDR 0 [0%] vs RDR 2 [2.7%], p= 0.47) | 1.3 | 0 |
| 2021 | Dai et al. | Early vs late | To test the hypothesis that early drain removal could decrease the incidence of grade 2 to 4 complications for patients undoing PD with low or intermediate risk of postoperative pancreatic fistula (POPF). | PPH (EDR 6 [3.8%] vs RDR 5 [3.2%], p= 0.75)PPH A (EDR 0 [0%] vs RDR 0 [0%])PPH B (EDR 6 [3.8%] vs RDR 5 [3.2%])PPH C (EDR 0 [0%] vs RDR 0 [0%])PPH MIPD (EDR 4 [10.3%] vs RDR 0 [0%], p= 0.17)PPH OPD (EDR 2 [1.7%] vs RDR 5 [4.1%], p= 0.47)PPH A MIPD (EDR 0 [0%] vs RDR 0 [0%])PPH A OPD (EDR 0 [0%] vs RDR 0 [0%])PPH B MIPD (EDR 4 [10.3%] vs RDR 0 [0%]) PPH B OPD (EDR 2 [1.7%] vs RDR 5 [4.1%]) PPH C MIPD (EDR 0 [0%] vs RDR 0 [0%])PPH C OPD (EDR 0 [0%] vs RDR 0 [0%]) | PPH: 3.5 PPH A: 0  PPH B: 3.5 PPH C: 0 PPH MIPD 5.4  PPH OPD 2.9  PPH A MIPD 0  PPH A OPD 0  PPH B MIPD 5.4  PPH B OPD 2.9  PPH C MIPD 0  PPH C OPD 0 | 0 |
| 2018 | Cecka et al. | Closed-suction drains vs passive gravity drains | To compare 2 types of intra-abdominal drains after pancreatic resection and their effect on the development of pancreatic fistulae and postoperative complications | Postoperative hemorrhage (closed-suction 9 [11%] vs passive gravity 15 [19%], p= 0.17) | 14.9 | - |
| 2014 | Jang et al. | Extended vs standard resection | To prospectively evaluate the survival benefit of dissection of the nerve plexus and lymphadenectomy in patients with pancreatic head cancer | Intrabdominal bleeding (SL n= 3 vs EL n= 5, no p-value) | 4.7 | 0 |
| 2016 | Sperling et al. | Extended vs standard resection | To evaluate whether ELA with the above specified locations is capable of improving progression-free survival compared to a standardized SLA | Bleeding requiring reoperation (SL n= 1, vs EL n= 2, p= 0.52) | 2.9 | - |
| 2017 | Ignjatovic et al. | Extended vs standard resection | To evaluate the benefit of extended lymphadenectomy PD and to estimate its impact on long-term survival | Early postoperative bleeding (SL 0% vs EL 3.3%, p> 0.05) | 1.6 | 33 |
| 2021 | Wang et al. | Extended vs standard resection | To determine the optimal lymphadenectomy extent for PD in the treatment of pancreatic head adenocarcinoma, we studied the effect of SPD and EPD on the survival time of patients | Intrabdominal bleeding (SL 6 [7.5%] vs EL 4 [5.4%], no p-value)GI bleeding (SL 4 [5%] vs EL 3 [4%], no p-value) | 11.1 | 60 |
| 2019 | Sabater et al. | Standard approach vs Artery-first approach | To compare the rates of R0 resection in PD for pancreatic and periampullary cancers by means of standard (ST) vs artery-first approach (AFA) | PPH (SA 8 [10.7%] VS AFA 8 [10.3%], p= 1) | 10.5 | - |
| 2014 | Tani et al. | Isolated Roux-en-Y vs conventional reconstruction | To compared the incidence of pancreatic fistula between the isolated Roux-en-Y(IsoRY) and conventional reconstruction (CR) methods | Intrabdominal hemorrhage (IsoRY 4 [5%] vs CR 1 [1%], p= 0.35) | 3.3 | 0 |
| 2014 | El Nakeeb et al. | Isolated Roux PJ vs Pancreaticogastrostomy | To compare the outcomes of isolated Roux loop pancreaticojejunostomy (IRPJ) with those of pancreaticogastrostomy (PG) after PD | Bleeding GJ (IRPJ n=1 vs PG n=1, p= 1)Bleeding PG (IRPJ n=0 vs PG n=2, p= 0.15)Internal hemorrhage (IRPJ n=1 vs PG n=1, p= 1) | 6.6 | - |
| 2012 | Uzunoglu et al. | Ultrasonic vs conventional | To assess the potential benefits of ultrasonic energy dissection compared with conventional dissection techniques in pancreatic surgery | PPH (ultrasonic 7 [12.5%] vs conventional 3 [6.4%], p= 0.36) | 9.9 | - |
| 2013 | Uzunoglu et al. | LigaSure vs Conventional | To compared the use of LigaSure with conventional dissection techniques in pancreatic surgery in a prospective randomized single-center trial | PPH (LigaSure n=4 vs Conventional n=4, p= 0.92) | 8.9 | - |
| 2020 | Gehrig et al. | LigaSure vs Conventional | To assess whether LigaSure Impact™ exhibits benefits over named conventional dissection techniques in patients undergoing PPPD | mITT analysisBleeding (LigaSure 1 [2%] vs Conventional 4 [10%], p= 0.15) PP analysis  Bleeding (LigaSure 0 [0%] vs Conventional 4 [14%], p= 0.056) | 6 | - |
| 2017 | Palanivelu et al. | Laparoscopic PD vs Open PD | To compare laparoscopic and open PD for short-term outcomes in a randomized trial | PPH (LPD n=4 vs OPD n=3, p= 0.39)  PPH A (LPD n=2 vs OPD n=1, no p-value)  PPH B (LPD n=1 vs OPD n=1, no p-value) PPH C (LPD n=1 vs OPD n=1, no p-value) | PPH: 10.9 PPH A: 4.6  PPH B: 3.1  PPH C: 3.1 | 0 |
| 2018 | Poves et al. | Laparoscopic PD vs open PD | To compare perioperative outcomes of PD performed through the laparoscopic route or by open surgery | Clinically relevant PPH (LPD 3 [9.3%] vs OPD 6 [20.7], p= 0.21) | 14.7 | 100 |
| 2019 | Van Hilst et al. | Laparoscopic PD vs open PD | To investigate if postoperative inflammatory markers differed between LPD and OPD and if there was a relationship between inflammatory markers and the occurrence of postoperative complications | PPH B/C (LPD n=2 vs OPD n=3, p> 0.99) | 13.1 | - |
| 2019 | Van Hilst et al. | Laparoscopic PD vs open PD | To assess whether laparoscopic PD could reduce time to functional recovery compared with open PD | PPH (LPD 5 [10%] vs OPD 7 [14%], p= 0.51)  PPH B (LPD 1 [2%] vs OPD 4 [8%], no p-value) PPH C (LPD 4 [8%] vs OPD 3 [6%], no p-value) | PPH: 12.1 PPH B: 5  PPH C: 7 | 50 |
| 2021 | Wang et al. | Laparoscopic PD vs open PD | To compare the outcomes of open PD (OPD) with those of LPD, when performed by experienced surgeons. | Modified intention to treat analysis:  PPH (LPD 37 [13%] vs OPD 33 [11%], p= 0.61)  PPH B/C (LPD 24 [8%] vs OPD 25 [8%], p= 0.88)  Per-protocol analysis:  PPH (LPD 35 [13%] vs OPD 24 [10%], p= 0.19) PPH B/C (LPD 22 [8%] vs OPD 17 [7%], p= 0.51) | mITT analysis:  PPH: 11.7  PPH B/C: 8.2  PP analysis  PPH: 11.4  PPH B/C: 7.5 | 36 |

*PD: pancreatoduodenectomy; DP: distal pancreatectomy; TP: total pancreatectomy; PJ: pancreaticojejunostomy; PG: pancreaticogastrostomy; PPPD:pylorus-preserving pancreatoduodenectomy; SSPPD: subtotal stomach-preserving pancreatoduodenectomy; BEE: Braun entero-enterostomy; MIDP: minimally-invasive distal pancreatectomy; LDP: laparoscopic distal pancreatectomy; ODP: open distal pancreatectomy; NPWT: negative pressure wound therapy; ERAS: enhanced recovery after surgery; EN: enteral nutrition; TPN: total parenteral nutrition; IAT: islet autotranplantation; PD: pancreatoduodenectomy; PPH: post-pancreatectomy hemorrhage; PJ: pancreaticojejunostomy; PG: pancreaticogastrostomy; mITT: modified intention-to-treat; HA: hepatic artery; GDA: gastroduodenal artery; PP: per-protocol; ETS: externalized trans-anastomotic stents; POPF: post-operative pancreatic fistula; CR-POPF: clinically relevant-postoperative pancreatic fistula; GI: gastrointestinal; DGE: delayed gastric emptying; TGJ: traditional gastrojejunostyomy; CS: circular stapler; HS: hand sewn; EDR: early drain removal; RDR: routine drain removal; SL: standard lymphadenectomy; EL: extended lymphadenectomy; SA: standard approach; AFA: artery-first approach; ISO-RY: isolated Roux-en-Y; CR: conventional reconstruction; IRPJ: isolated roux loop pancreaticojejunostomy*
