# Supplementary material for: Treatment strategies to prevent or mitigate the outcome of postpancreatectomy hemorrhage: a review of randomized trials
Source: Int J Surg. 2023 Nov 16;110(10):6145–54. doi: 10.1097/JS9.0000000000000876 (PMC11486935; doi:10.1097/JS9.0000000000000876)
Supplement: SUPPLEMENTARY MATERIAL [file js9-110-6145-s005.docx]

## **Supplementary table 3. Results of RCTs on distal pancreatectomy**

| **Year** | **Authors** | **Topic** | **Primary aim** | **PPH Results** | **Total PPH  rate (%)** | **Mortality  PPH related (%)** |
| --- | --- | --- | --- | --- | --- | --- |
| 2012 | Hamilton et al. | Mesh reinforcement vs no-mesh reinforcement | To compare stapled left pancreatectomy with stapled left pancreatectomy using mesh reinforcement of the staple line with either Seamguard or Peristrips Dry | Post-operative bleeding requiring transfusion:  Mesh (POF n=0 vs no POF n=0, no p-value) No mesh (POF n=1 vs no POF n=1, no p-value)  Postoperative bleeding requiring reoperation:  Mesh (POF n=0 vs no POF n=1, no p-value) No mesh (POF n=1 vs no POF n=0, no p-value)  GI bleed:  Mesh (POF n=0 vs no POF n=0, no p-value) No mesh (POF n=1 vs no POF n=0, no p-value) | 5 | - |
| 2019 | Kondo et al. | Reinforced stapler vs bare stapler | To investigate whether reinforced staplers reduce the incidence of clinically relevant pancreatic fistula (PF) after DP compared with staplers without reinforcement | PPH (RS 0 [0%] vs SS 2 [3.4%], p= 0.08) | 1.6 | 0 |
| 2021 | Wennerblom et al. | Reinforced stapling vs standard stapling | To evaluate the effect of staple bio-reinforcement on the frequency of POPF after DP | PPH (RS 1 [2%] vs SS 2 [4%], p= 0.48) | 2.8 | 0 |
| 2014 | Antila et al. | Roux-Y Binding PJ vs hand-sewn | To investigate whether finnish binding PJ FBPJ is feasible and prevents the risk for POPF even after left pancreatectomy | PPH (Roux-Y Binding n=0 vs hand-sewn n=0) | 0 | 0 |
| 2016 | Kawai et al. | PJ vs stapling closure | To evaluate in a multicenter RCT whether PJ of pancreatic stump decreases the incidence of pancreatic fistula after DP compared with stapler closure. | Intrabdominal hemorrhage (PJ 0 [0%] vs staple 2 [3.3%], p= 0.26) | 1.6 | 0 |
| 2017 | Uemura et al. | Duct-to-mucosa PG vs hand-sewn closure | To investigate whether duct-to-mucosa PG of the pancreatic stump decreased clinical POPF formation compared with handsewn closure after DP | Intrabdominal hemorrhage (PG n=1 vs hand-sewn n=0, p= 0.49) | 1.3 | - |
| 2016 | Hassenpflug et al. | Teres ligament patch vs no teres ligament patch | To analyze the impact of teres ligament covering on pancreatic fistula rate after DP. | PPH (Patch 5 [6.6%] vs no patch 8 [10.5%], p= 0.22) | 8.6 | 0 |
| 2012 | Montorsi et al. | Tachosil vs no Tachosil | To evaluate the role of an absorbable fibrin sealant patch (TachoSil) in reducing POPF after DP | Hemorrhage in hospital (Tachosil 1 [1%] vs no Tachosil 3 [2%], no p-value)Hemorrhage 2 months FU from discharge (Tachosil 1 [1%] vs no Tachosil 1 [1%], no p-value) | 2.1 | 0 |
| 2015 | Sa Cunha et al. | Tachosil vs no Tachosil | To evaluate the effectiveness of TachoSil sponge on DP remnant stump in reducing the rate and severity of POPF | Intra-abdominal bleeding (Tachosil n=5 vs no Tachosil n=4, no p-value) | 3.3 | 0 |
| 2016 | Park et al. | Tachosil vs no Tachosil | To investigate the clinical outcomes, including POPF, afterusing the TachoSil patch in DP | Postoperative bleeding (Tachosil 1 [2.1%] vs no Tachosil 0 [0%], p= 0.29) | 1 | 0 |
| 2021 | Mungroop et al. | Tachosil vs no Tachosil | To investigate whether an absorbable fibrin sealant patch could prevent POPF after DP | PPH (patch 2 [1.6%] vs no patch 6 [4.9%], p= 0.17) | 3.2 | - |
| 2017 | Van Buren et al. | Drain vs no drain | To test the hypothesis that DP without intraperitoneal drainage does not affect the frequency of grade 2 or higher-grade complications | Intra-abdominal bleeding (drain 2 [1%] vs no drain 2 [1%], p= 0.98) GI bleeding (drain 2 [1%] vs no drain 0 [0%], p= 0.49) | 1.7 | 0 |
| 2020 | Dai et al. | Early vs late | To assess whether early drain removal after major pancreatectomy influences the incidence of complications in the patients with low risk of postoperative pancreatic fistula (POPF) | Postoperative hemorrhage (EDR 0 [0%] vs RDR 2 [2.7%], p= 0.47) | 1.3 | 0 |
| 2018 | Cecka et al. | Passive drain vs closed-suction drain | To compare 2 types of intra-abdominal drains after pancreatic resection and their effect on the development of pancreatic fistulae and postoperative complications | Postoperative hemorrhage (passive 9 [11%] vs closed-suction 15 [19%], p= 0.17) | 14.9 | - |
| 2021 | Yamada et al. | Separate division vs combined division | To establish the safety of combined division of the splenic vein compared with separate division of the splenic vein | Intrabdominal hemorrhagePPH A (separate 0 [0%] vs combined 0 [0%], no p-value)PPH B (separate 0 [0%] vs combined 1 [0.6%], no p value)PPH C (separate 2 [1.3%] vs combined 1 [0.6%], no p-value)All (separate 2 [1.3%] vs combined 2 [1.3%], p> 0.99) | PPH: 1.2 PPH A: 0  PPH B: 0.3 PPH C: 0.9 | 0 |
| 2021 | Landoni et al. | Stapled vs ultrasonic transection | To compare stapled versus ultrasonic transection in elective distal pancreatectomy. | PPH (stapled 3 [4%] vs ultrasonic 8 [11%], p= 0.10) | 8 | 100 |
| 2019 | De Rooij et al. | MIDP vs ODP | To compare time to functional recovery after minimally invasive and open DP | PPH (LDP 2 [4%] vs 2[4%], p> 0.99)PPH B (LDP 2 [4%] vs 1[2%], no p-value)PPH C (LDP 0 [0%] vs 1[2%], no p-value | PPH: 3.7 PPH B: 2.7 PPH C: 0.97 | - |
| 2020 | Bjornsson et al. | LDP vs ODP | To compare short-term surgical outcomes following LDP and ODP with the hypothesis that laparoscopy would shorten hospital stay in patients undergoing standard DP | PPH (LDP n=1 vs ODP n=0, p= 0.31)PPH A (LDP n=1 vs ODP n=0, no p-value)PPH B (LDP n=0 vs ODP n=0, no p-value)PPH C (LDP n=0 vs ODP n=0, no p-value | PPH: 1.7 PPH A: 1.7  PPH B: 0 PPH C: 0 | 0 |

*DP: distal pancreatectomy; PJ: pancreaticojejunostomy; PG: pancreaticogastrostomy; GI: gastrointestinal; POPF: post-operative pancreatic fistula; PPH: post-pancreatectomy hemorrhage; RS: reinforced stapler; SS: standard stapler; RCT: randomized controlled trial; EDR: early drain removal; RDR: routine drain removal; MIDP: minimally-invasive distal pancreatectomy; LDP: laparoscopic distal pancreatectomy; ODP: open distal pancreatectomy;*
