# Supplementary material for: Treatment strategies to prevent or mitigate the outcome of postpancreatectomy hemorrhage: a review of randomized trials
Source: Int J Surg. 2023 Nov 16;110(10):6145–54. doi: 10.1097/JS9.0000000000000876 (PMC11486935; doi:10.1097/JS9.0000000000000876)
Supplement: SUPPLEMENTARY MATERIAL [file js9-110-6145-s006.docx]

**Supplementary table 4. Results of RCTs on various perioperative interventions.**

| Year | Authors | Topic | Primary aim | PPH Results | Total PPH  rate (%) | Mortality  PPH related (%) |
| --- | --- | --- | --- | --- | --- | --- |
| 2018 | Yamamoto et al. | 1-day antimicrobial prophylaxis vs 5-days prophylaxis | To determine the optimal duration of antimicrobial prophylaxis in patients treated with PD who underwent preoperative biliary drainage (PBD) but were without cholangitis | PPH (1-day 0 [0%] vs 5-day 1 [2%], p= 0.24) | 1.2 | 0 |
| 2020 | Andrianello et al. | PICO vs standard sterile dressing | To compare negative pressure wound therapy with standard sterile dressing in terms of the prevention of non-organ-space surgical site infection (superficial and deep surgical site infection) in the high-risk setting | PPH B/C (PICO 7 [15.2%] vs SSD 3 [6.1%], p= 0.19)PPH B (PICO 6 [13%] vs SSD 2 [4.1%], p= 0.28) PPH C (PICO 1 [2.2%] vs SSD 1 [2%], no p-value) | PPH: 10.5 PPH B: 8.4  PPH C: 2.1 | - |
| 2020 | Singh et al. | Bile clamping vs no bile clamping | To assess the impact of intra-operative bile duct clamping on surgical site infection (SSI) following PD | PPH (Clamping 2 [10%] vs no clamping 1 [5%], p= 1) | 7.5 | - |
| 2020 | De Pastena et al. | Wound protector vs standard drape | To assess whether a wound protector could reduce the risk of superficial incisional SSI after PD | PPH (WP 15 [16%] vs SD 14 [15%], p= 0.47) | 15.3 | - |
| 2017 | Deng et al. | mERAS vs no ERAS | To investigate the feasibility and safety of implementing the ERAS protocol in patients undergoing PD | Bleeding (mERAS n=6 vs no ERAS n=4, p= 0.5) | 6.2 | 0 |
| 2019 | Hwang et al. | ERAS vs no ERAS | To investigate the non-inferiority of Enhanced Recovery After Surgery (ERAS) for PD | PPH B/C (ERAS 2 [1.6%] vs no ERAS 4 [3.2], no p-value) | 2.4 | 0 |
| 2019 | Takagi et al. | ERAS vs no ERAS | To examine the efficiency of ERAS protocols in patients following PD | Hemorrhage (ERAS 1 [3%] vs no ERAS 1 [3%], p= 1) | 2.7 | 0 |
| 2019 | Hou et al. | ERAS vs no ERAS | To explore the effect of accelerated rehabilitation program on the prognosis of pancreatic cancer patients undergoing pancreaticoduodenectomy. | Hemorrhage (ERAS 1 [1.9] vs traditional 4 [7.6], no p-value) | 9.6 | 0 |
| 2021 | Ergenc et al. | ERAS vs no ERAS | To investigate the effect of the ERAS protocol on post-operative complications, LOS, and readmission rates in pancreatic surgery patients | PPH (ERAS n=2 vs no ERAS n=4, p= 0.66) | PPH: 15.7 PPH A:10.5  PPH B: 5.2  PPH C: 0 | 0 |
| 2015 | Van Samkar et al. | Crystalloid fluid restriction vs standard fluid therapy | To investigate effects of crystalloid fluid restriction in pancreatic surgery. Our hypothesis: enhanced recovery of gastrointestinal function | PPH (restricted 2 [6%] vs standard 3 [9%], p= 0.67) | 7.5 | - |
| 2017 | Weinberg et al. | Goal directed therapy vs standard fluid therapy | To evaluate perioperative outcomes in patients undergoing PD with or without a cardiac output goal directed therapy (GDT) algorithm. | GI bleeding (GDT 0 [0%] vs standard fluid therapy 2 [8%], p> 0.99) | 3.8 | 0 |
| 2016 | Zhang et al. | Preoperative Ulinastatin vs no Ulinastatin | To investigate the efficacy of ulinastatin on pancreatic fistula and other complications after PD | Hemorrhage (Ulinastatin 3 [7%] vs no Ulinastatin 2 [4%], P-value not significant) Hemorrhage (POPF 5 [11%] vs no POPF 0 [0%], p= 0.051) | 5.4 | 0 |
| 2020 | Bergeat et al. | Nasogastric tube up to 3-5 days vs no nasogastric tube | To assess in a randomized clinical trial whether the absence of systematic NGTD after PD reduces postoperative complications | PPH (NGT 9 [15.3%] vs no NGT 3 [5.8%], p= 0.19) | 10.8 | 37.5 |
| 2022 | Smith et al. | Intraoperative secretin vs standard therapy | To identify leaks and subsequently target operative intervention would decrease the frequency of clinically significant post-operative pancreatic fistula formation | Hemorrhage mild (secretin n=2 vs no secretin n=1, no p-value)hemorrhage moderate (secretin n=1 vs no secretin n=4, no p-value) Hemorrhage Severe (secretin n=6 vs no secretin n=10, no p-value) | Total: 14.1Mild: 1.7 Moderate2.9  Severe: 9.4 | 0 |
| 2022 | Jeong et al. | Preemptive albumin administration vs standard therapy | To investigate the effect of preemptive and empirical albumin administration to prevent fluid overload and edema on postoperative outcomes in patients undergoing pancreatectomy | Postoperative hemorrhage (Albumin n=8 vs no albumin n=4, p= 0.37) | 6 | - |
| 2022 | Smits et al. | PORSCH vs Standard | To design a multimodal algorithm for the early recognition and minimally invasive management of postoperative complications in patients having pancreatic resection for all indications | PPH requiring intervention (PORSCH 47[5%] vs standard 51[6%], p=0.046 | 5.6 | - |
| 2013 | Belyaev et al. | Intraoperative intra-arterial octreotide vs no octreotide | To test the hypothesis that intra-arterial application of octreotide in the gastroduodenal artery during pancreatectomy may increase pancreatic hardness. | Hemorrhage (Octreotide 2 [16.7%] vs no octreotide 1 [7.7%], p= 0.59) | 12 | 100 |
| 2018 | El Nakeeb et al. | Postoperative octreotide vs standard | To evaluate the effect of postoperative use of octreotide on the outcomes after PD | Bleeding PG (Octreotide 0 [0%] vs no octreotide 1 [1.9%], p= 0.31)  Internal hemorrhage (Octreotide 1 [1.9%] vs no octreotide 1 [1.9%], p= 1) | 2.8 | 0 |
| 2021 | Cao et al. | Prophylactic somatostatin vs no somatostatin | To assess the preventive efficacy of somatostatin on clinically relevant POPF in intermediate-risk patients who underwent PD at pancreatic centers in China | Late postoperative hemorrhage (Somatostatin 7 [7%] vs standard 12 [12%], p= 0.23)  Late postoperative hemorrhage OPD (Somatostatin 6 [10%] vs standard 12 [17%], no p-value)  Late postoperative hemorrhage LPD (Somatostatin 1 [2%] vs standard 0 [0%], no p-value) | Total: 9.5  OPD: 14.1  LDP: 1.3 | 0 |
| 2016 | Laaninen et al. | Perioperative hydrocortisone vs placebo | To study whether post-PD complications (PPDC) in high-risk patients can be reduced with hydrocortisone. | PPH (Hydrocortisone 14% vs placebo 24%, p= 0.35) Major complication (hydrocortisone 18% vs placebo 41%, P < 0.05) | PPH 12.9 | 0 |
| 2020 | Tarvainen et al. | Pasireotide vs Hydrocortisone | To assess the non-inferiority of hydrocortisone compared with pasireotide in reducing complications after partial pancreatectomy | PPH (Pasireotide 0 [0%] vs Hydrocortisone 7 [11%], p= 0.01)  PPH B/C (Pasireotide 0 [0%] vs Hydrocortisone 6 [10%], p= 0.01)  PD:  Any PPH (Pasireotide 0 [0%] vs Hydrocortisone 4 [15%], p= 0.044; PPH B/C Pasireotide 0 [0%] vs Hydrocortisone 4 [15%], p= 0.044)  DP: Any PPH (Pasireotide 0 [0%] vs Hydrocortisone 3 [10%], p= 0.23;  PPH B/C Pasireotide 0 [0%] vs Hydrocortisone 2 [7%], p= 0.49). | PPH 5.5  PPH A: 0.7  PPH B/C: 4.7 | 0 |
| 2013 | Zhu et al. | Polyunsaturated fatty acid diet vs no polyunsaturated fatty acid diet | To investigate the effect of parenteral fish oil lipid emulsion in parenteral nutrition (PN) supplementation combined with enteral nutrition (EN) support on PD | Intraperitoneal bleeding (PUFA 2 [5.3%] vs no PUFA 1 [2.6%], no p-value)  Digestive bleeding (PUFA 1 [2.6%] vs no PUFA 2 [5.3%], no p-value) | 7.8 | 0 |
| 2014 | Aida et al | Preoperative oral immunonutrition vs standard nutrition | To investigate the effect of preoperative IN on operative complications, and the participation of prostaglandin E2 (PGE2) on T-cell differentiation in patients undergoing a severely stressful surgery | Intra-abdominal bleeding (IN n=1 vs standard n=1, p= 1) | 4 | - |
| 2012 | Park et al. | EN vs TPN | To assess the postoperative nutritional status of patients who had undergone PD according to the postoperative nutritional method between EEN and TPN, and compared the clinical outcomes of the two modes | Postoperative bleeding (EN n= 0 VS TPN n= 1, P= 0.36) | 2.6 | 0 |
| 2016 | Perinel et al. | EN vs TPN | To compare nasojejunal early enteral nutrition (NJEEN) with TPN, after PD | PPH (EN 25 [31.6%] vs TPN 24 [36.9%], p= 0.50)  PPH B/C (EN 22 [21.6%] VS TPN 22 [21.8], P= 0.97) | PPH: 24  PPH A: 2.5  PPH B/C: 21.5 | 7.6 |
| 2019 | Wu et al. | Enteral feeding vs oral feeding | To test the hypothesis that oral feeding is non-inferior to enteral feeding in closure of POPF after PD, and to clarify the effects of oral feeding on the duration and grade of POPF | PPH (Enteral n=0 vs oral n= 2, p= 0.49) | 1.7 | 0 |
| 2021 | Liu et al. | Early nasojejunal feeding vs early oral feeding vs saline | To test the hypothesis that early oral feeding (EOF) is superior to early nasojejunal nutrition (ENN) after pylorus-preserving PD(PPPD) in terms of delayed gastric emptying (DGE). | PPH (nasojejunal 1 [2.5%] vs oral 1 [2.5%] vs 2 [5%], p= 1) | 3.3 | 0 |

*PPH: post-pancreatectomy hemorrhage; PBD: preoperative biliary drainage; NPWT: negative pressure wound therapy; PD: pancreatoduodenectomy; DP: distal pancreatectomy; ERAS: enhanced recovery after surgery; GDT: goal direct therapy; GI: gastrointestinal; NTG: naso-gastric tube; POPF: post-operative pancreatic fistula; MIDP: minimally-invasive distal pancreatectomy; LDP: laparoscopic distal pancreatectomy; ODP: open distal pancreatectomy; EN: enteral nutrition; PN: parenteral nutrition; TPN: total parenteral nutrition; PUFA: polyunsaturated fatty acid;*
